# Treatment of Incontinence of Urine by Vesical Dilatation

**Published:** 1885-09

**Authors:** 


### Treatment of Incontinence of Urine by Vesical Dilatation.

Dr. Felipe Margarit reports, in the *Gæsta Medical Catalana*, a case of incontinence of urine in a boy 12 years old. All medication had failed. At first only an ounce of water could be forced into the bladder, but the capacity of the organ gradually increased by daily injections, till it would hold seven ounces at the end of a month. As the capacity of the bladder increased, the ability to retain the urine increased also.

